# Postimplantation syndrome following stentless thoracic endovascular aortic repair of chronic aortic dissection

**DOI:** 10.1016/j.jvscit.2025.101933

**Published:** 2025-07-22

**Authors:** Justin Blackman, Brandon McGuinness, Vamshi K. Kotha

**Affiliations:** a Island Medical Program, Faculty of Medicine, University of British Columbia, University of Victoria, Victoria, British Columbia, Canada; b Department of Vascular Surgery, Vancouver Island Health Authority, Victoria, British Columbia, Canada; c South Island Medical Imaging, Vancouver Island Health Authority, Victoria, British Columbia, Canada

**Keywords:** Postimplantation syndrome, TEVAR, Endovascular repair, Aortic dissection, Systemic inflammatory response, Corticosteroids, Stentless

## Abstract

Postimplantation syndrome (PIS) is a noninfectious systemic inflammatory response that often follows endovascular aneurysm repair, historically presumed to be a reaction to implanted graft materials. Although well-documented with stent graft use, PIS in the absence of stent grafts remains poorly understood. We present an instance of PIS after stentless thoracic endovascular aortic repair, which involves embolizing intimal tears with plugs and/or coils. This case highlights the need for further investigation into the pathophysiology of PIS. PIS can occur in the absence of a stent graft; prophylactic anti-inflammatory therapy may be warranted during endovascular interventions with large-volume aneurysms or false lumen thrombosis.

Postimplantation syndrome (PIS) is a noninfectious postoperative systemic inflammatory response characterized by pyrexia, elevated white blood cell (WBC) count, and elevated C-reactive protein (CRP); it is observed in approximately 40% of endovascular aneurysm repair (EVAR) cases with a range of clinical severity.^1^^,^^2^ PIS has been theorized as the body's response to the foreign graft composition.^3^^,^^4^ However, the exact etiology has not yet been elucidated, and endothelial disruption^5^ and systemic response to rapid thrombus formation^6^ may be contributing factors. We report a case of stentless thoracic endovascular aortic repair (TEVAR), wherein only embolization coils and a single vascular plug were used, which resulted in PIS in the absence of an endograft. The inflammatory response observed suggests that there are triggers for PIS beyond graft composition.

## Case report

**Fig 1:**
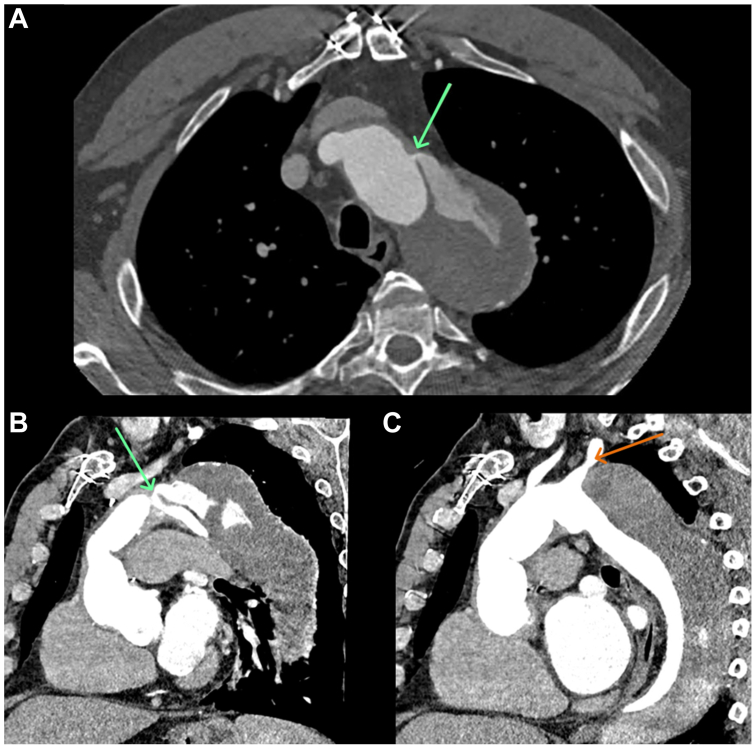
Preprocedural contrast-enhanced electrocardiogram-gated computed tomography (CT) scan of the thoracic aorta. **(A)** Axial image demonstrating the small intimal tear providing antegrade perfusion of the false lumen (*green arrow*). **(B)** Left anterior oblique multiplanar reconstruction image of the aortic arch demonstrating the same antegrade perfusion of the false lumen (*green arrow*). **(C)** Another left anterior oblique multiplanar reconstruction image of the aortic arch demonstrating the position of the aneurysmal deformity relative to the left subclavian artery (*orange arrow*).

A 62-year-old man with a prior surgical aortic root and hemi-arch repair for Stanford type A dissection that extended to zone 10 was found to have a gradually enlarging proximal descending thoracic aorta (zones 3-5) 7 years postoperatively. This finding was related to persistent false lumen perfusion from a small intimal tear in the aortic arch (zone 2), adjacent to the left subclavian artery. The zone 4 total aortic diameter measured 7.5 cm (Fig 1). The patient had chronic atrial fibrillation, chronic kidney disease, hypertension, and hyperlipidemia. He experienced a perioperative stroke during the prior aortic repair. Repeat open surgery was considered high risk, and standard TEVAR would have required debranching of the aortic arch. The patient declined surgery. Given that the primary intimal tear measured only 3 mm on computed tomography (CT) angiogram, a stentless TEVAR approach was offered and accepted by the patient. An 8-mm plug (Amplatzer Vascular Plug 4; Abbott Vascular) was selected to close the intimal tear with 167% oversizing. This dumbbell-shaped device straddles the dissection flap, occluding the intimal tear. When fully deployed, each half measures approximately 8.0 × 6.5 mm, avoiding subclavian ostial occlusion. The patient did not have coronary artery disease; his preoperative WBC count was 8.3 × 10^9^/L.

**Fig 2:**
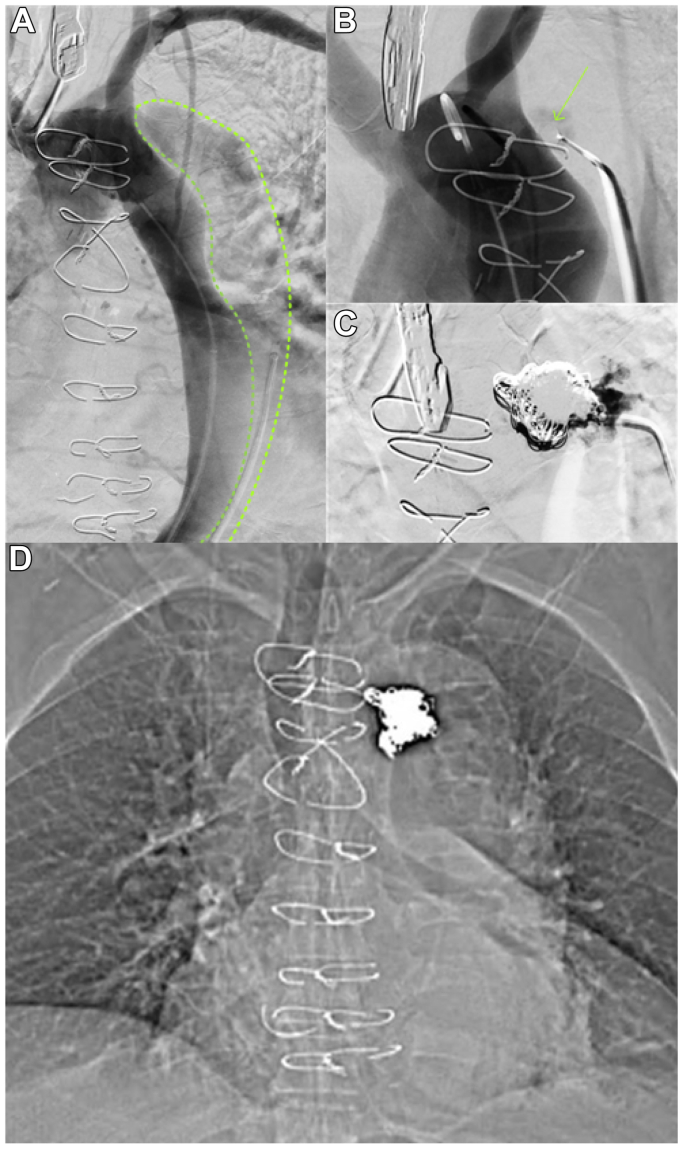
Procedural imaging of stentless thoracic endovascular aortic repair (TEVAR). **(A)** Selective angiogram via a pigtail catheter within the true lumen shows communication to the false lumen (*green dashes*) before repair. **(B)** Selective angiogram via a pigtail catheter within the true lumen shows reduced antegrade perfusion into the false lumen after placement of the vascular plug (*green arrow*). **(C)** Selective angiogram via the Rim catheter within the false lumen shows no communication between the lumens following placement of embolization coils. **(D)** Coronal unenhanced computed tomography (CT) scan demonstrating positioning of the vascular plug and embolization coil pack.

The stentless TEVAR (Fig 2) proceeded with bilateral common femoral artery access. A pigtail catheter was placed in the true lumen of the aortic arch. The false lumen was accessed through a distal intimal tear in the left common iliac artery using a 5.0F Rim catheter under angiographic guidance, with a glidewire advanced and its position within the false lumen at the arch confirmed by transesophageal echocardiography. A single 8-mm plug was deployed under transesophageal echocardiography and angiographic guidance. Nonfibered embolization coils (Ruby Coil System, Penumbra) were deployed in the false lumen adjacent to the plug. Rapid thrombosis of the false lumen was observed. Postoperative imaging demonstrated adequate positioning with no residual false lumen flow. The patient was discharged on the same day in good health.

Five days later, the patient presented to the emergency department complaining of fever, rigors, and a witnessed brief loss of consciousness. Routine hematology revealed leukocytosis, monocytosis, and neutrophilia. Blood cultures grew coagulase-negative *Staphylococcus* in only one of two simultaneously collected sets; this result was retrospectively attributed to contamination, given the organism's frequent contamination of cultures^7^ and the absence of growth on repeat cultures despite worsening symptoms. The patient was ultimately admitted on broad-spectrum antibiotic therapy. CT angiogram after admission demonstrated an unchanged plug position with no antegrade false lumen perfusion through the repair.

The patient did not improve on empiric broad-spectrum antibiotic therapy. The patient continued to experience fever and rigors with fluctuating WBC counts. A total of 13 investigations for infectious etiology, including blood cultures, urine cultures, serology, and molecular microbiology panels, were conducted across seven different dates during the patient's hospitalization, all of which were negative.

**Fig 3:**
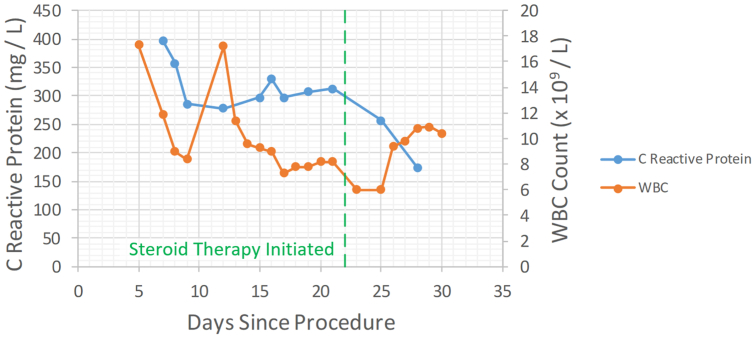
Select inflammatory marker laboratory values for the patient while admitted. *WBC*, white blood cell.

**Fig 4:**
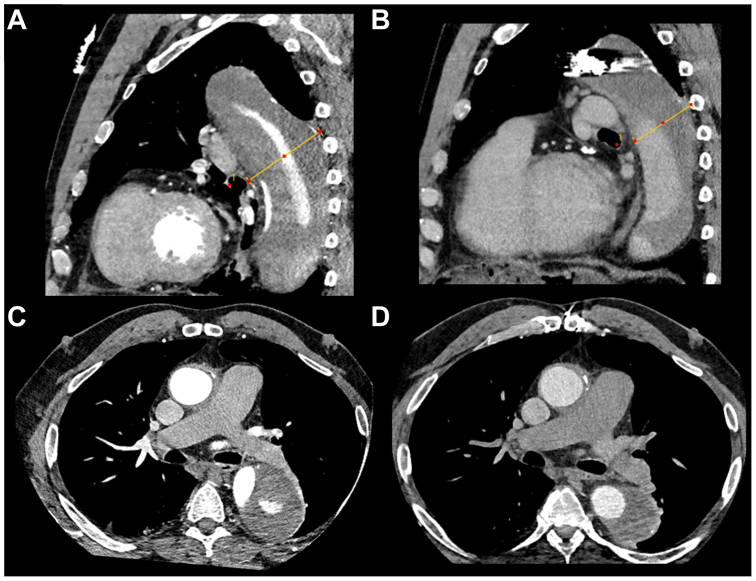
Side-by-side comparison of preprocedural and 12-month postprocedural contrast-enhanced electrocardiogram-gated computed tomography (CT) images, demonstrating aortic remodeling. **(A)** Preprocedural sagittal reconstruction of zone 4 aorta at maximum diameter (75.1 mm). **(B)** Postprocedural sagittal reconstruction of zone 4 aorta at maximum diameter (55.6 mm). **(C)** Preprocedural axial image of the zone 4 aorta. **(D)** Postprocedural axial image of the zone 4 aorta.

A trial of a nonsteroidal anti-inflammatory drug was initiated on postprocedural day 17. The patient's clinical presentation immediately improved with the cessation of fever, chills, and rigors, but his CRP remained elevated (Fig 3). Steroid therapy was initiated on postprocedural day 24, consisting of a 3-day course of stress-dose intravenous hydrocortisone followed by a maintenance dose of oral prednisone. The patient's CRP and WBC count both returned to normal. The patient was discharged on postprocedural day 30, with steroid dose tapering instructions. Antibiotic therapy continued throughout admission and after discharge, for a total of a 6-week course, out of an abundance of caution. A WBC scan on postprocedural day 47 demonstrated physiologic distribution of radiotracer activity with no evidence of progressive white cell accumulation in the excluded aneurysm or surrounding the embolic devices. The patient was followed with repeat contrast-enhanced electrocardiogram-gated CT of the aorta at 1, 2, 6, and 12 months post-discharge. These scans showed ongoing positive aortic remodeling with progressive reduction in false lumen size and zone 4 aortic diameter decreasing from 75 mm to 56 mm (Fig 4). The patient made a full recovery without further complications related to his aortic procedure. This individual case report was considered a medical/educational activity not meeting the definition of research under TCPS2 and did not require research ethics board approval. Written informed consent was obtained from the patient for publication of this case report and the accompanying images.

## Discussion

PIS can occur after arterial interventions, even with minimal use of implants. Although PIS after a frozen elephant trunk procedure has been reported previously,^8^ it has not been reported in the absence of any graft material. This case, along with previous reports of significant inflammatory responses after arterial embolization^9^, ^10^, ^11^ suggests that endothelial disruption and thrombus formation, rather than graft composition alone, are involved in triggering PIS. However, it remains possible that the new thrombus may be a consequence of the systemic inflammatory response, rather than its cause. Given the potential severity of inflammatory response, there may be a role for prophylactic anti-inflammatory therapy during stentless TEVAR cases or endovascular interventions with a large volume of expected false lumen thrombosis. A single-center, randomized, double-blind, placebo-controlled trial of 30 mg/kg of methylprednisolone administered preoperatively before EVAR, involving 153 adult patients between 2009 and 2013, showed a significant decrease in the incidence of systemic inflammatory response syndrome and shortened time to discharge, but did not impact readmission rates.^12^

The possibility of PIS should be considered in cases of systemic inflammatory response after endovascular intervention, particularly when associated with large-volume thrombosis. Commonly proposed diagnostic criteria for PIS include fever of greater than 38.0°C, WBC count of greater than 12 × 10^9^/L, and CRP of greater than 10 mg/L in the absence of infection.^12^^,^^13^ Although generally self-limited, major organ dysfunction after an inflammatory response to EVAR has been reported, and PIS is associated with significantly higher 30-day mortality and major adverse cardiac events.^12^^,^^14^^,^^15^ There is currently no universally accepted treatment for moderate or severe PIS.^5^ Reported approaches have included 50 mg of intravenous hydrocortisone administered four times daily and 100 mg of oral nimesulide once daily.^15^ In this case, after failure of symptom resolution with naproxen, treatment with 50 mg of intravenous hydrocortisone twice daily for 3 days, followed by 20 mg of oral prednisone once daily for 1 week, was successful. In light of the benefits observed with prophylactic high-dose steroid therapy,^12^ a moderate regimen such as 10 mg of intravenous dexamethasone administered perioperatively could plausibly confer similar advantages and warrants consideration for further investigation.

## Conclusions

PIS can occur even in the absence of a stent graft. There may be a role for prophylactic anti-inflammatory therapy during endovascular interventions with expected large volume aneurysms or false lumen thrombosis.

## Availability of data and materials

Data sharing is not applicable to this article as no datasets were generated or analyzed during the current study. Further information can be made available by the authors on request.

## Funding

None.

## Disclosures

None.
